# Changes in Behavior and Neural Dynamics across Adolescent Development

**DOI:** 10.1523/JNEUROSCI.0462-23.2023

**Published:** 2023-12-13

**Authors:** Lucrezia Liuzzi, Daniel S. Pine, Nathan A. Fox, Bruno B. Averbeck

**Affiliations:** ^1^Emotion and Development Branch, National Institute of Mental Health, Bethesda, 20892, MD; ^2^Department of Human Development and Quantitative Methodology, University of Maryland, College Park, MD 20742; ^3^Laboratory of Neuropsychology, National Institute of Mental Health, Bethesda, 20892, MD

**Keywords:** adolescence, attractor, dynamics, computational modeling, development, EEG, pruning

## Abstract

Adolescence is an important developmental period, during which substantial changes occur in brain function and behavior. Several aspects of executive function, including response inhibition, improve during this period. Correspondingly, structural imaging studies have documented consistent decreases in cortical and subcortical gray matter volume, and postmortem histologic studies have found substantial (∼40%) decreases in excitatory synapses in prefrontal cortex. Recent computational modeling work suggests that the change in synaptic density underlie improvements in task performance. These models also predict changes in neural dynamics related to the depth of attractor basins, where deeper basins can underlie better task performance. In this study, we analyzed task-related neural dynamics in a large cohort of longitudinally followed subjects (male and female) spanning early to late adolescence. We found that age correlated positively with behavioral performance in the Eriksen Flanker task. Older subjects were also characterized by deeper attractor basins around task related evoked EEG potentials during specific cognitive operations. Thus, consistent with computational models examining the effects of excitatory synaptic pruning, older adolescents showed stronger attractor dynamics during task performance.

**SIGNIFICANCE STATEMENT** There are well-documented changes in brain and behavior during adolescent development. However, there are few mechanistic theories that link changes in the brain to changes in behavior. Here, we tested a hypothesis, put forward on the basis of computational modeling, that pruning of excitatory synapses in cortex during adolescence changes neural dynamics. We found, consistent with the hypothesis, that variability around event-related potentials shows faster decay dynamics in older adolescent subjects. The faster decay dynamics are consistent with the hypothesis that synaptic pruning during adolescent development leads to stronger attractor basins in task-related neural activity.

## Introduction

Adolescence involves substantial changes in brain and behavior (Benasich and Ribary, 2018; Blakemore, 2018; Gee et al., 2018; Nussenbaum and Hartley, 2019). Neuroimaging work has shown that gray matter volume decreases during adolescence, while white matter increases (Giedd, 2004; Bethlehem et al., 2022). Histologic analysis of postmortem tissue has also shown that excitatory synapses in cortex, which primarily occur on spines, peak between ages 5 and 10, and then decrease exponentially into the mid-20s (Huttenlocher, 1979; Huttenlocher and Dabholkar, 1997; Petanjek et al., 2011). Some studies have found that up to 40% of synapses are pruned during adolescence (Petanjek et al., 2011). Furthermore, synaptic density decreases earlier in primary sensory areas than in association areas (Huttenlocher and Dabholkar, 1997; Elston et al., 2009). Examination of resting-state oscillations in EEG and MEG have also found that low-frequency oscillations decrease during adolescence, and high-frequency oscillations increase (Whitford et al., 2007; Segalowitz et al., 2010; Candelaria-Cook et al., 2022; Rempe et al., 2023). These changes in oscillations correlate with developmental changes in gray matter and white matter (Candelaria-Cook et al., 2022). Evoked responses also develop over adolescence, with early sensory-motor peaks decreasing in amplitude and in peak latency. Other evoked responses associated with cognitive control, performance monitoring and working memory, change in both morphology and location, like the P3 response seen in go/no-go tasks and the error-related negativity (ERN) response, both increasing in amplitude over the adolescent years (Jonkman, 2006; Segalowitz et al., 2010; Tamnes et al., 2013). However, few studies use measures of brain function to test mechanistic theories related to synaptic pruning, and the current report provides such mechanistic data.

Behavior undergoes substantial change during adolescence in ways that could relate to pruning. Although basic perceptual abilities reach adult levels by late childhood (Leat et al., 2009), executive processing, such as working memory and inhibitory control, depend on prefrontal cortex (Goldman-Rakic, 1996) and continue to develop into adulthood (Larsen and Luna, 2018; Theodoraki et al., 2020; Ferguson et al., 2021). Reinforcement learning (RL), which is the ability to learn to select actions that are rewarding and avoid actions that are harmful (B.B. Averbeck and Costa, 2017; B. Averbeck and O'Doherty, 2022), also improves (Lange-Küttner et al., 2012, 2021; Nussenbaum and Hartley, 2019). While RL is often thought to be driven by subcortical plasticity, recent work suggests that working memory may also play an important role in RL (Collins and Frank, 2012; Yoo and Collins, 2022). Cognitive control, measured with speeded response tasks like the Eriksen Flanker task, also improves during adolescence (Luna et al., 2010).

Few mechanistic theories link changes in behavior during adolescence with changes in the brain. It has often been assumed that maturation of prefrontal cortex influences behavior (Caballero et al., 2016). This, however, leaves open the question of mechanisms that link prefrontal cortex maturation to changes in behavior. Recently, a computational model was developed that explicitly linked pruning in recurrent cortical networks to improvements in behavior (B.B. Averbeck, 2022). This study trained recurrent neural networks on working memory and RL tasks. It was found that, if recurrent connections in the network were pruned during training, networks performed better than networks that were given the same amount of training, but not pruned. The current study tests this theory using longitudinal data in children and adolescents.

In simulated data, pruning led to working memory networks that were more resistant to distraction, and RL networks that learned to select rewarding options more consistently, similar to what is seen during adolescent development (Nussenbaum and Hartley, 2019). Furthermore, the pruning led to a characteristic signature in the neural dynamics. Specifically, a metric known as the Lyapunov exponent decreased with pruning. These decreases relate to changes in attractor dynamics and resistance to network perturbation. Thus, pruned neural networks have smaller Lyapunov exponents and stronger attractor dynamics than unpruned networks. Although the network models were trained to reproduce behavior, the pruning led to consistent changes in the neural dynamics, and it is the changes in the neural dynamics that we examine in the present manuscript.

We sought to test the hypothesis that adolescent development, and the corresponding pruning that takes place, leads to stronger attractors during task performance, and therefore measurable changes in dynamics. To do so, we fit dynamical systems models to EEG data collected longitudinally in a large cohort of human subjects, at 12, 15, and 18 years of age. At each age point the subjects performed the Eriksen Flanker task while EEG data were collected. Consistent with model predictions, we found that time-varying linear estimates of the neural dynamics were consistent with stronger dynamical attractors in older subjects. We also found that these changes in dynamics correlated with improvements in reaction time.

## Materials and Methods

### Data

Children were recruited for a larger longitudinal study at the University of Maryland assessing behavior (Fox et al., 2001) and EEG markers of error response over development (Buzzell et al., 2017; Filippi et al., 2020). Participants included in this analysis were selected as they completed a Flanker task with EEG at age 12, 15, and/or 18 years. Not all subjects were sampled at all time points.

We preprocessed a total of 484 EEG recordings from 213 participants. We included time locked data with at least 50 trials passing our quality assessment criteria giving us a clean sample of 324 stimulus locked and 307 cue locked and response locked datasets. The clean time locked datasets came from 331 separate recording sessions from 179 children (104 female, 75 male). The average ages at the three recording time points were 13.1 (SD 0.5), 16.0 (SD 0.5), and 18.4 (SD 0.6) for the 12-, 15-, and 18-year target age groups, respectively.

The task consisted of 384 trials separated into 12 blocks. At the end of each block, the computer presented feedback based on the child's performance. In blocks where accuracy was 90% or above, the feedback was “Respond faster.” In blocks where accuracy was between 75% and 90%, the feedback was “Good job.” And in blocks where accuracy was below 75%, the feedback was “Be more accurate.” This feedback was designed so that children produced a sufficient number of errors.

Each trial consisted of presentation of a fixation cross visual cue (lasting ∼300–600 ms), followed by a visual stimulus composed of five horizontally aligned arrowheads presented for 200 ms. The stimulus was followed by a blank screen for ∼1900 ms.

Participants were instructed to indicate as quickly as possible with a button press the direction of the central arrow. The task had four stimulus types appearing with equal probability: two congruent stimuli where all five arrows pointed in the same direction (⋘≪, ⋙≫), and two incongruent stimuli with the “flanking” arrows pointing in the opposite direction from the central arrow (≪>≪, ≫<≫).

A 128-channel HydroCel Geodesic Sensor Net and EGI software (Electrical Geodesic) were used for all data collection. Data were sampled online at 250 Hz and referenced to the vertex electrode Cz. Data were converted to EEGLAB format before preprocessing trough the MADE pipeline (Debnath et al., 2020). Preprocessing included deleting the outer cap electrodes with the largest artefacts (going from 128 to 104 electrodes), eliminating 1s data segments with noise spikes, high-pass filtering at 0.2 Hz, lowpass filtering at 50 Hz, cleaning of eye motion artefacts with Independent Components Analysis (ICA) with the ADJUST toolbox (Mognon et al., 2011), segmenting the data with reference to the cue, stimulus, and motor response, and finally referencing all sensors from Cz to the electrode average. The full preprocessing code is available on GitHub, and all preprocessing and subsequent analyses were conducted with MATLAB 2020a (The MathWorks) and EEGLAB toolbox v.2022.0 (Delorme and Makeig, 2004).

### Analysis

The event-related potential (ERP) spectral density is dominated by low frequencies (<10 Hz). To focus on the evolution of the evoked response components we low pass filtered the EEG data at 15 Hz and down sampled to 30 Hz. All trials in which participants responded to the stimulus were included (i.e., all correct and commission error trials) in both congruent and incongruent conditions.

Our goal was to fit linear dynamical systems models to examine the dynamical properties of the EEG signals around the mean responses. We considered four task conditions: congruent versus incongruent, and correct versus commission errors, crossed. There was no evidence that left/right responses were coded in the EEG signal (*p* > 0.05) so we did not break-out the response type as separate conditions. To reduce the spatial dimensionality of our EEG data we applied principal components analysis (PCA). We calculated the PCA coefficients on the time concatenated stimulus locked and response locked conditions (on the grand-averaged data from all age groups). The PCs were estimated by computing a covariance matrix across these vectors such that the dimensionality of the covariance matrix was 104 × 104 (given by the number of sensors). We then extracted PCs from this matrix. We selected the first two components for the analysis of the dynamics. By extracting the PCs from the average ERPs, we were targeting a low-dimensional representation in the space of the task-related signals. Note that the PCs have no a-priori theoretical relationship with our task, or with the predictions of the computational model. They were used as a signal processing tool to identify a low-dimensional space of task-related evoked activity in which we could test our hypothesis.

**Figure 1.**
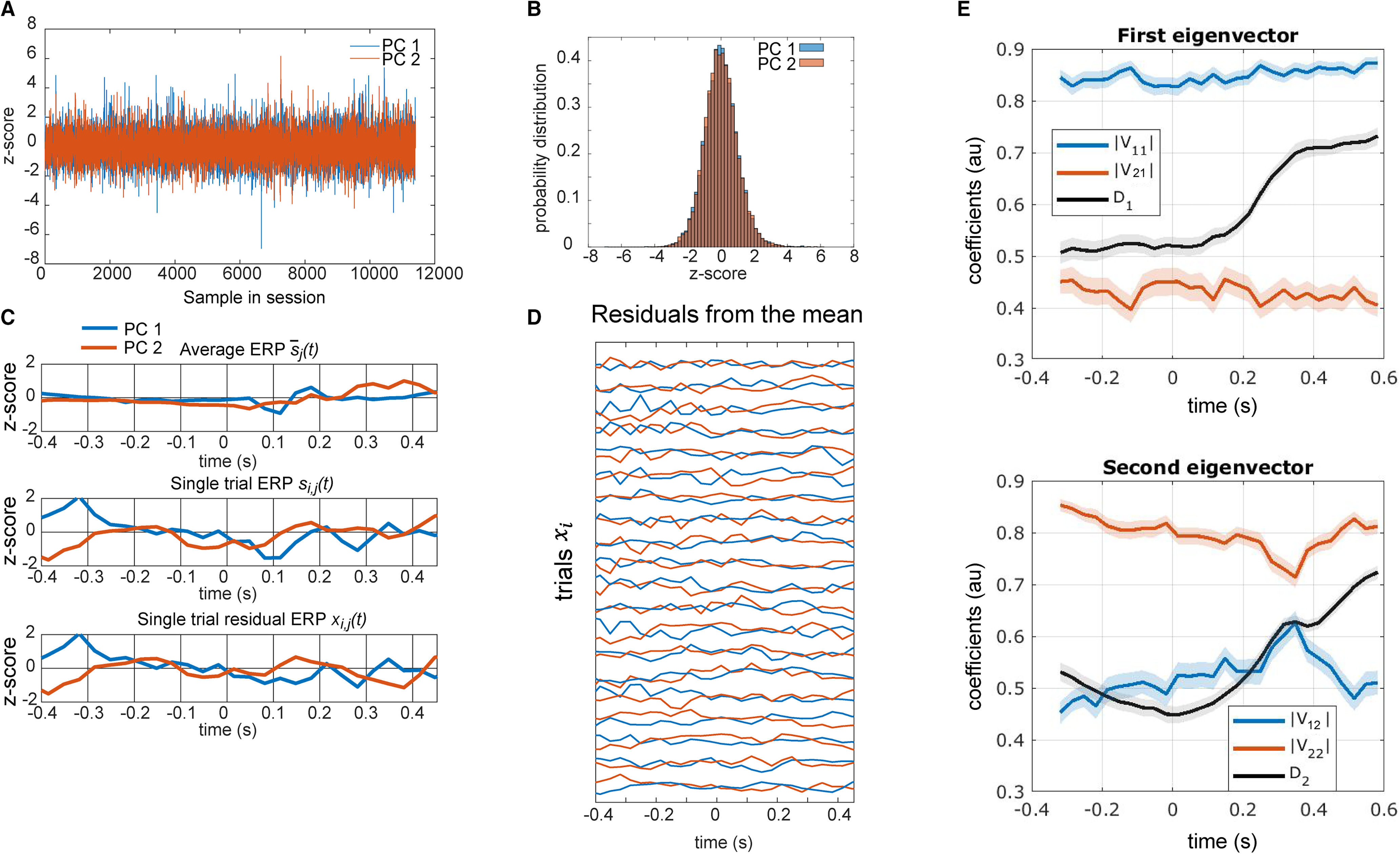
Dynamical systems analysis steps. ***A***, Activity from the 104 sensors was first projected into the first two PCs for each subject. This panel shows the time series across a session for a single subject in PC space after z-scoring. ***B***, Distribution of activity for a single subject after z-scoring the first two PCs, showing that data has a zero mean and SD of 1. ***C***, Top, Mean response for a single subject for a single condition in the two PCs. Middle, Single trial evoked response. Bottom, Residual after subtracting the mean response for the corresponding condition from the evoked response. ***D***, Residual activity from a series of trials for a single subject. ***E***, Average and SEM (shaded region) across subjects of the coefficients from the eigenvectors extracted from the A matrix. V is the eigenvector matrix. The first value V_11_ corresponds to the coefficient related to the first principal component and the second value V_12_ corresponds to the coefficient related to the second principal component. The variable D_1_ is the eigenvalue that corresponds to this eigenvector. Bottom, Same analysis applied to the second eigenvector.

In the next step we projected the data from each time point in each trial into the PC space. This reduced the dimensionality from the 104 sensors to two PC dimensions. We then z-scored each PC for each subject (Fig. 1*A*). The z-score was computed by first computing the mean and SD across all time points for each component, and then using these to standardize the data. Thus, we subtracted one overall mean from each channel and divided by the overall SD. The resultant time-series in each PC had a mean of 0 and a SD of 1 (Fig. 1*B*).

Data were next segmented in windows around the presentation of the fixation cue (−600–500 ms relative to the cue), presentation of the flanker stimuli (−400–600 ms relative to the flanker stimuli), and the subject's response (−500–500 ms relative to button responses) for each trial. The dynamics analysis characterizes the temporal evolution (i.e., relaxation) of fluctuations around the mean evoked activity $\bar{s}(t)$, where *t* is time in trial. We assessed the relaxation back to the mean response $\bar{s}(t)$ by estimating the decay of the single trial residuals in the 2D PCA space. The residual (Fig. 1*C*,*D*), $x_{i,j}(t)$, is equal to the difference between the single trial activity $s_{i,j}(t)$, where *i* is trial and *j* is condition, and the average across trials for that condition $\bar{s_{j}}(t)$, i.e., $x_{i,j}(t)=s_{i,j}(t)-\bar{s_{j}}(t)$. Our analyses were then fit to this residual activity (Galgali et al., 2023).The dynamical systems models characterize the rate at which noise in the residual activity decays back to the mean.

We next fit an autoregressive (AR1), linear model to the 2D residual neural activity, x(t), where we have dropped the subscripts for trial and condition to simplify notation:

x(t+1)=Ax(t) + ε(t).

The variable ε(t) is the noise term, assumed to be independent identically distributed (i.i.d.) Gaussian, and A is the 2 × 2 dynamics matrix, where the two dimensions correspond to the two PCs. The variable x(t) is correspondingly a 2D vector of the residual activity at time *t*. The model was fit to data points in a 100 ms moving window (at 30 Hz, this corresponds to three time points from each trial sampled every ∼33 ms). Thus, data points in a 100-ms window, relative to the trial event being considered (e.g., the cue), from all trials were pooled and used to fit the model. This resulted in a time-dependent estimate of the matrix *A*. We then extracted the eigenvalues of matrix *A* and subjected them to further random effects analyses to assess our hypothesis. Eigenvalues of A closer to 0 indicate a faster decay and eigenvalues near 1 would correspond to a slower decay. We expect the eigenvalues of A to be between 0 and 1, and to be smaller in older adolescents.

### Statistical tests

According to our hypotheses we expect the eigenvalues of the matrix A to show modulation with the behavioral task and to have smaller values in older adolescents (i.e., a negative relationship between age and eigenvalues). We first tested the eigenvalues with the analysis window locked to each task condition (cue, stimulus, and response) for effects of age. To account for multiple EEG recordings belonging to the same children at different ages we used a linear mixed effect model with a random effect of participant:

eigenvalue=age + (1|participant)+1

We conducted a similar analysis to examine the effect of sex with and without age:

eig=sex + (1|participant)and eig=age + sex + (1|participant).

We also tested the hypothesis that the eigenvalues of A would relate to task performance, with smaller eigenvalues being related to shorter reaction times (positive relationship) and higher task accuracy (negative relationship). Again, to account for the longitudinal recordings we tested these hypotheses with linear mixed effects models:

RT=eigenvalue + (1|participant) + 1

Accuracy=eigenvalue + (1|participant) + 1.

Mixed effects models were run independently for each eigenvalue and time window. To account for multiple comparisons over time, we applied a one dimensional temporal clustering model with the threshold free cluster enhancement algorithm on the fixed effects *t* statistics (Smith and Nichols, 2009). The null distribution for the temporal cluster was obtained by bootstrap sampling the fixed effect variable for all time points, obtaining the *t* statistics of the mixed effect model on the bootstrapped time course, applying temporal clustering and picking the highest (or lowest, depending on test hypothesis) cluster value for that time course. The bootstrap procedure was repeated 10,000 times for each hypothesis test to obtain a null distribution for the temporal clusters. For the tests of age effects on eigenvalues we conducted tests locked to three different task epochs (cue, stimulus, and response) and 2 eigenvalues and specific hypotheses on the direction of the relationships. Therefore, we selected one-tailed Bonferroni corrected significance tests with α-level of 0.05/6 = 0.0083. For the analysis of behavioral effects, we conducted nine tests on 2 eigenvalues. Therefore, we selected one-tailed Bonferroni corrected significance tests with α-level of 0.05/18 = 0.0027.

### Analysis of time-jittered datasets

To generate datasets with jittered ERPs in single trials, we generated single session data, where each trial's evoked response $s^{b}{}_{i,j}\left(t\right)$, where the superscript indicates jittered data, was given by:

$$s^{b}{}_{i,j}\left(t\right)=\bar{s_{j}}\left(t+\tau \right)+\varepsilon (t).$$

For each trial, τ was sampled from a mean 0 Gaussian distribution where the SD of the Gaussian was given by the average SD for the corresponding age group. The variable ε(t) indicates zero mean Gaussian noise with a SD given by the average across all subjects and groups.

## Results

**Figure 2.**
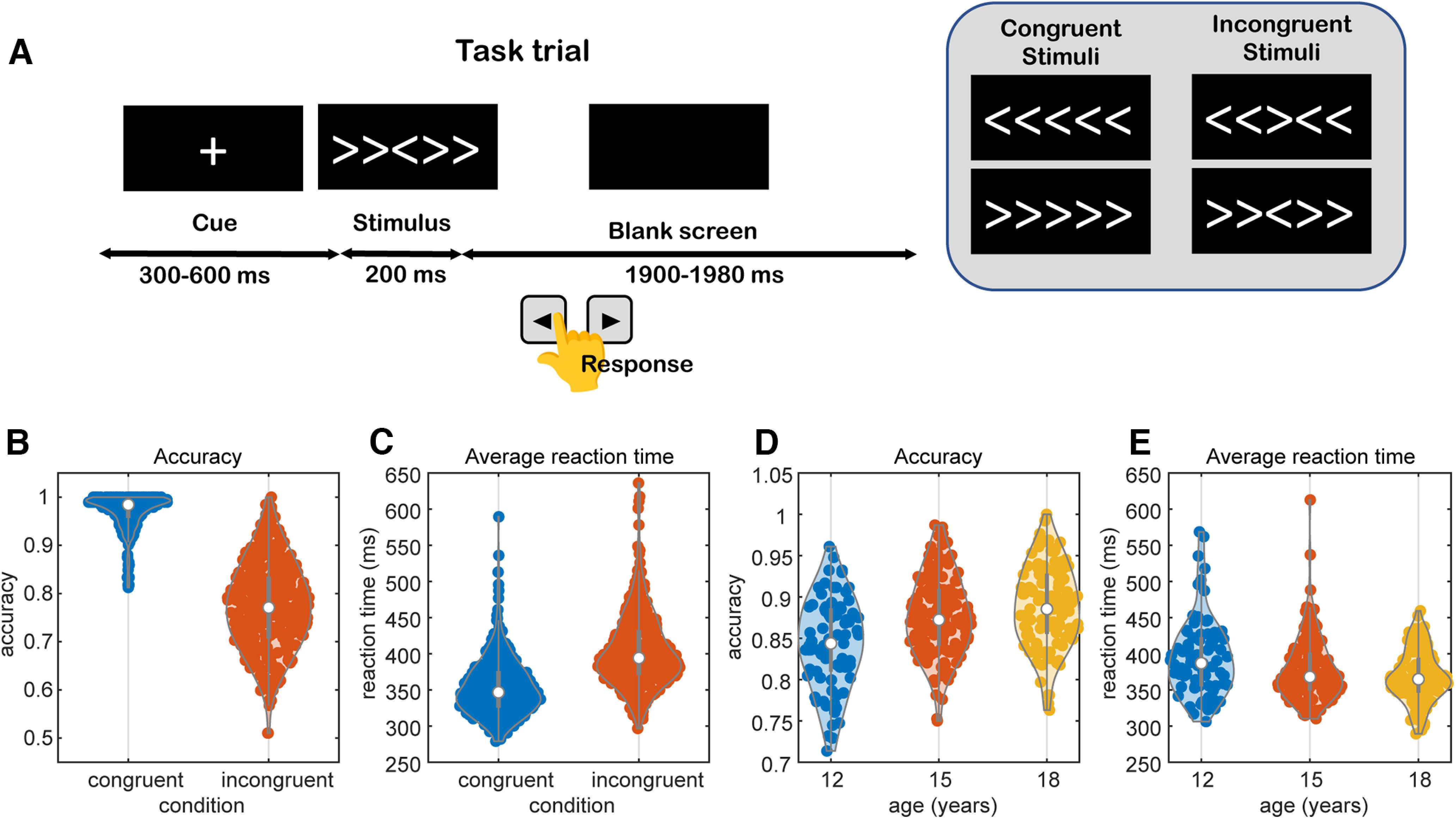
Task and behavioral results. ***A***, In each trial, subjects were first shown a cue at the center of the screen. After a variable delay, a stimulus was shown, and the subjects were required to indicate whether the central arrow was pointing left or right as quickly and accurately as possible. ***B***, Response accuracy by condition, averaged across age groups. ***C***, Reaction time by condition, averaged across age groups. ***D***, Accuracy by age, averaged across conditions. ***E***, Reaction time by age, averaged across condition.

We conducted 128 channel EEG recordings in adolescents while they executed the flanker task. Data were collected at three ages longitudinally for a total of 331 recordings from 179 adolescents (after quality control). In the task, subjects executed 384 trials in 12 blocks in a single session. Subjects were first shown a cue (plus sign), and after a variable delay, they were given a stimulus (Fig. 2*A*). Their task was to indicate with a key press whether the central arrow pointed left or right. The task had congruent and incongruent conditions. In the congruent condition all arrows pointed in the same direction, and in the incongruent condition the flanking arrows pointed in the opposite direction of the central arrow. At the end of each block, subjects were given feedback which was “good job,” “be more accurate,” or “go faster,” to keep their accuracy between 75% and 90% correct. We found that participants were more accurate [congruent accuracy 0.97 (SD 0.04), incongruent 0.77 (SD 0.10), paired-sample *t*_(330)_ = 41.3, *p* < 0.001], and faster [RT congruent 354 ms (SD 40 ms), incongruent 404 ms (SD 52 ms), *t*_(330)_ = 42.2, *p* < 0.001] in congruent than incongruent trials. When we examined the effect of age on behavior, we found that accuracy increased with age [Fig. 2*B*; *F*_(1,329)_ = 54.72, *p* < 0.001), and reaction times decreased with age (Fig. 2*C*; *F*_(1,329)_ = 35.32, *p* < 0.001]. Thus, subjects became more proficient at the task over adolescent development. We also found a small effect of sex on task performance with males responding faster [males RT 369 ms (SD 41 ms), females 387 ms (SD 47 ms), unpaired-sample *t*_(329)_ = 3.6, *p* < 0.001)] and with slightly less accuracy [males 0.86 (SD 0.06), females 0.88 (SD 0.05), unpaired-sample *t*_(329)_ = 2.5, *p* = 0.01)].

**Figure 3.**
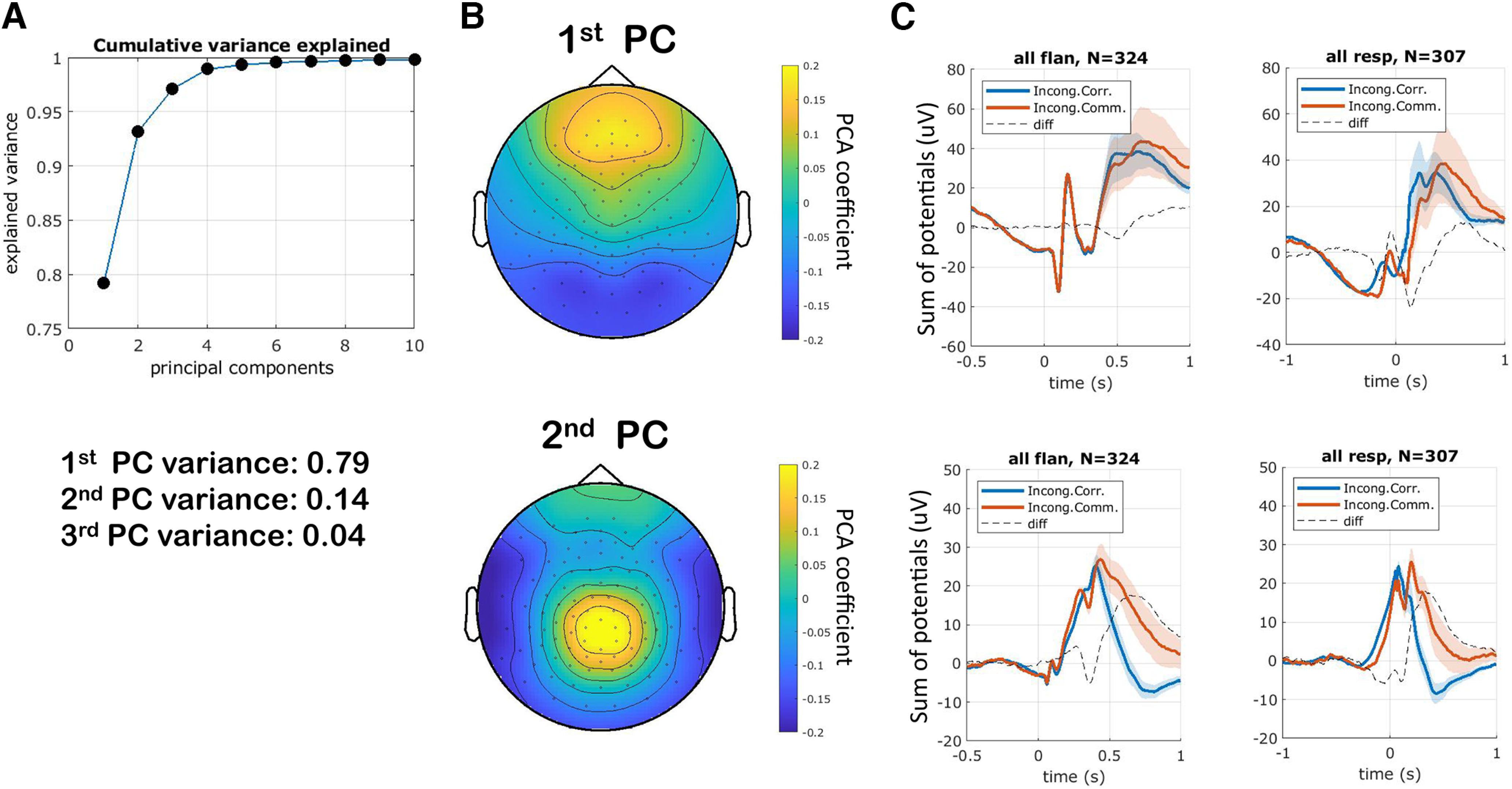
PCA analysis. Data dimensionality was reduced by applying PCA to the grand average evoked responses. ***A***, The first principal component explained 79% of the variance. ***B***, Spatial maps of principal components. ***C***, Subject average ERPs projected into PCA space for incongruent correct and error trials locked to either the stimulus (flan) or the response (resp).

We began the analysis of the EEG data by calculating a set of principal components in sensor space. The components were based on the average response in each condition (concatenated), with the average taken across all trials and subjects (Fig. 3; see Materials and Methods). The first component was a frontal and occipital component explaining 79% of the variance, while the second was a central/parietal and temporal component explaining 14% of the variance (Fig. 3*A*). The second component also captured most of the differences between correct and error responses. Component 1 reflected an anterior-posterior gradient and component 2 was more centrally focused (Fig. 3*B*). The first PC shows a rapid negative response, at around 100 ms, and may reflect visual inputs (Fig. 3*C*). The second PC separates correct responses from commission errors (i.e., an incorrect button press), especially a few hundred milliseconds after the response (Fig. 3*C*).

To begin the analysis of the dynamics, the data for individual trials in which subjects made a response were projected into the first two PCs (see Materials and Methods; Fig. 1). The data in each PC was then z-scored within each subject using the mean and SD across all trials and time points. This standardized the variance across subjects and age groups. We then computed the time-varying mean of the z-transformed data for each condition and subtracted the mean for each condition for all trials of that condition. This resulted in the residual activity, around the mean, for each single trial. This was done for both PCs.

We then fit a 2D linear dynamical system model to the residual neural activity in the two PC dimensions in 100-ms moving windows (Materials and Methods). The eigenvalues of the linear dynamical system, extracted from the matrix *A* (Materials and Methods) characterize the dynamics of the neural activity, around the mean neural activity. The first eigenvalue primarily reflected the first PC and the second eigenvalue primarily reflected the second PC, except around 375 ms after stimulus onset. Small eigenvalues indicate fast dynamics, in which variability in neural activity rapidly relaxes back to the mean, and larger eigenvalues indicate slower dynamics, in which variability in neural activity relaxes slowly back to the mean. A value of 1 would be a perfect integrator, a condition in which the activity would integrate variability instead of relaxing back to the mean, as in a diffusion process. Our hypothesis is that adolescent development will lead to faster dynamics, and therefore eigenvalues will get smaller with age. This follows from computational modeling in recurrent neural networks, looking at the effects of pruning on working memory and reinforcement learning (B.B. Averbeck, 2022).

To test this hypothesis, we examined age-dependent changes in the time-dependent eigenvalues. The dynamics models were fit in 100-ms windows time locked to cue onset, stimulus onset, or response onset. We used a moving window analysis under the assumption that the dynamics would change over time. We also assumed that they would vary relative to different events in the task.

**Figure 4.**
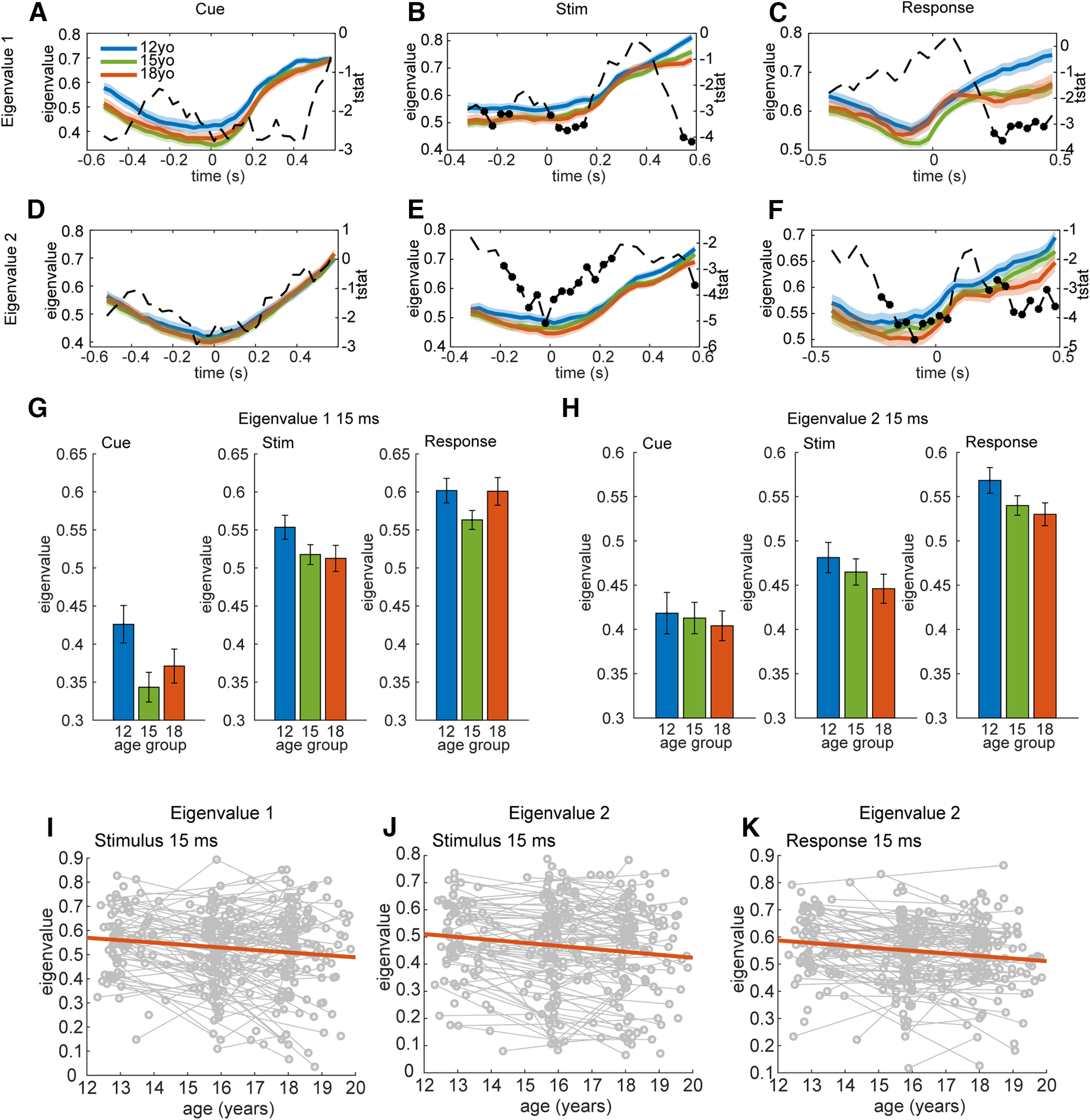
Linear effect of age on cue, stimulus, and response locked data. ***A***, Eigenvalue 1 versus time for cue locked data. The fixed effect of the model eigenvalue ∼ age + 1|subject is shown as a dashed black line with significant datapoints (*p* < 0.05/6 cluster corrected) as asterisks. ***B***, Eigenvalue 1 versus time for stimulus locked data. ***C***, Eigenvalue 1 versus time for response locked data. ***D–F***, Same as ***A–C*** for eigenvalue 2. ***G***, Bar plots showing the effects of age group on eigenvalue 1 at 15 ms after each task event. ***H***, Same as ***G*** for eigenvalue 2. ***I***, Within subject scatter plot of age effect on the first eigenvalue 15 ms after presentation of the stimulus. J. Within subject scatter plot of age effect on second eigenvalue 15 ms after presentation of the stimulus. ***K***, Same as ***J***, 15 ms after the response.

We found significant effects of age for both eigenvalues in the stimulus locked and response locked analyses. Consistent with specificity in age-related changes, we found no significant effects in the cue locked analyses (Fig. 4*A*,*D*). The first eigenvalue showed a significant linear effect of age in the stimulus locked condition from 252 to 152 ms before the stimulus, and 15 to 148 (Fig. 4*G*,*I*) and 548 to 581 ms after the stimulus (Fig. 4*B*). The first eigenvalue also showed significant effects in the response locked condition from 248 to 448 ms after the response (Fig. 4*C*). The second eigenvalue showed significant effects in the stimulus locked analyses from 185 ms before to 248 ms after the stimulus (Fig. 4*E*,*H*,*J*). There were also significant effects of age in the response locked analyses from 219 ms before to 48 ms after the response (Fig. 4*H*,*K*), and 215–481 ms after the response (Fig. 4*F*). Overall, the effects in the second eigenvalue were more widespread in time than the first eigenvalue, but they were locked to specific events. Our model predicts that changes in neural dynamics will be task related. It does not, however, make specific predictions in specific tasks about which epochs would shows changes. However, given the specificity of our effects, our results cannot be accounted for by global changes in the frequency spectrum with age.

Given the known difference in the onset of puberty in males and females we performed exploratory analyses including sex as predictor of differences in the eigenvalues. Over all six conditions (2 eigenvalues and three time-locked conditions) and time-points we found a maximum effect of sex for the first eigenvalue in the cue condition with *t*_(305)_ = 2.59 (*p* = 0.01), which did not survive correction for multiple comparisons (0.05/6 = 0.0083 Bonferroni corrected.)

**Figure 5.**
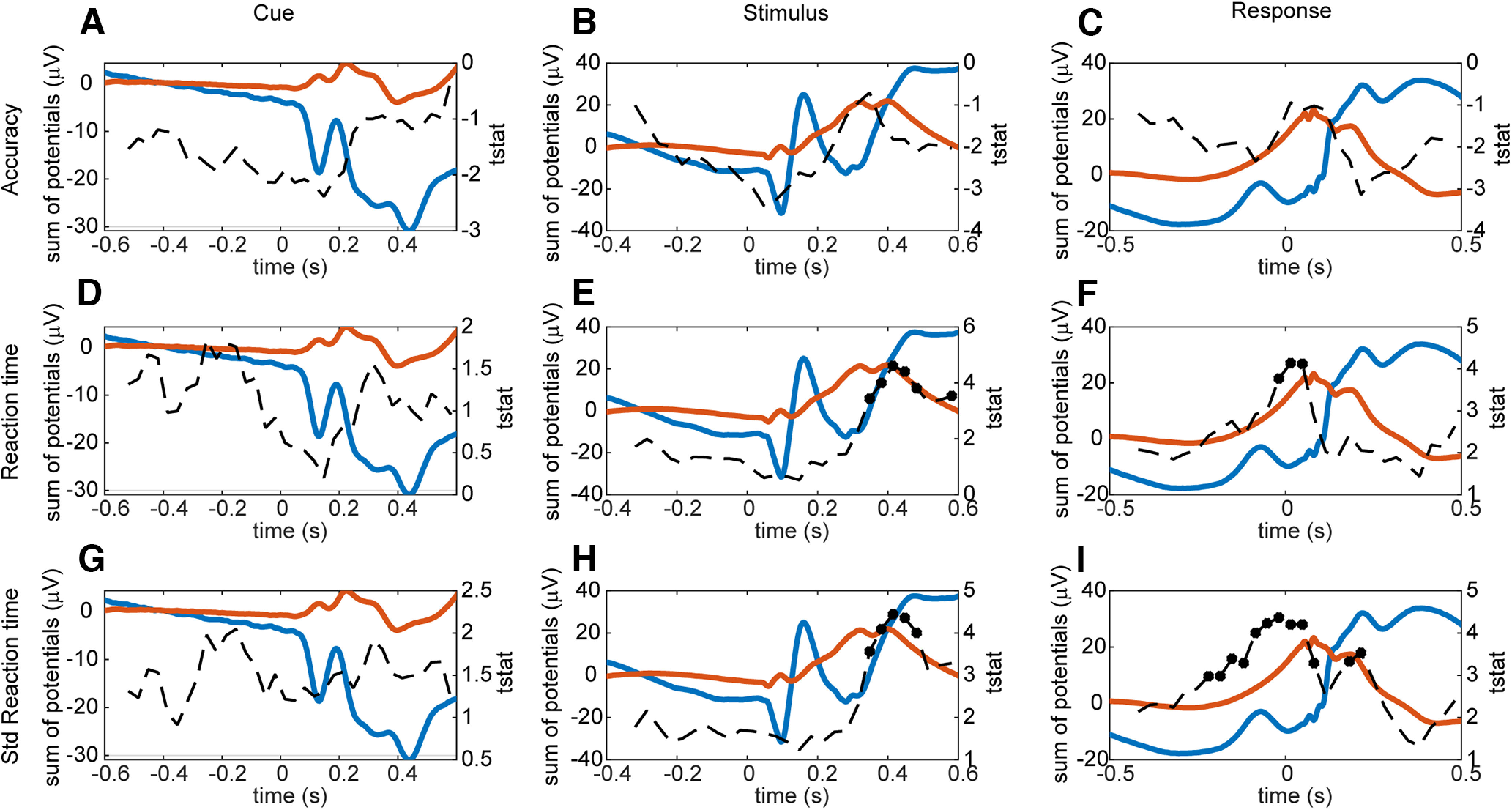
Linear effects of the second eigenvalue on subject's accuracy, mean reaction time and SD of reaction time over trials. Red and blue lines show the first and second PC of the ERP averaged across conditions, correspondingly, and dashed line is the time-dependent *t* statistic. ***A***, Cue locked analysis of correlation between accuracy and eigenvalue. ***B***, Stimulus locked analysis of correlation between accuracy and the second eigenvalue. ***C***, Response locked analysis of correlation between accuracy and eigenvalue. ***D–F***, Same as ***A–C*** for correlation between reaction time and the second eigenvalue. ***G–I***, Same as ***A–C*** for correlation between the SD of the reaction time and the second eigenvalue.

We next examined correlations between eigenvalues and, behavior including accuracy, reaction times, and the SD of reaction time (Fig. 5). There were no behavioral correlations with the first eigenvalue, across subjects that survived correction for multiple comparisons. In the cue locked analyses, we also found no correlations between eigenvalues and any of the behavioral measures (Fig. 5*A*), similar to the analyses of age. In the stimulus locked analysis, we found correlations between eigenvalues and reaction times (Fig. 5*E*) and the SD of the reaction time (Fig. 5*H*), from 348 to 481 ms after the stimulus (peak at 415 ms, effect estimate 0.233 (SD 0.051) for mean RT and 0.247 (SD 0.056) for RT SD). This is near the average reaction time (Fig. 2*C*). We also found correlations between the eigenvalues and the reaction time at the time of the response [Fig. 5*F*; from 19 ms before to 48 ms after the response; peak at 15 ms after, effect estimate 0.227 (SD 0.055)] and the SD of the reaction time from 219 ms before until 81 ms and 181–215 ms after the response [Fig. 5*I*, peak at −0.19 ms, effect estimate 0.255 (SD 0.058)].

**Figure 6.**
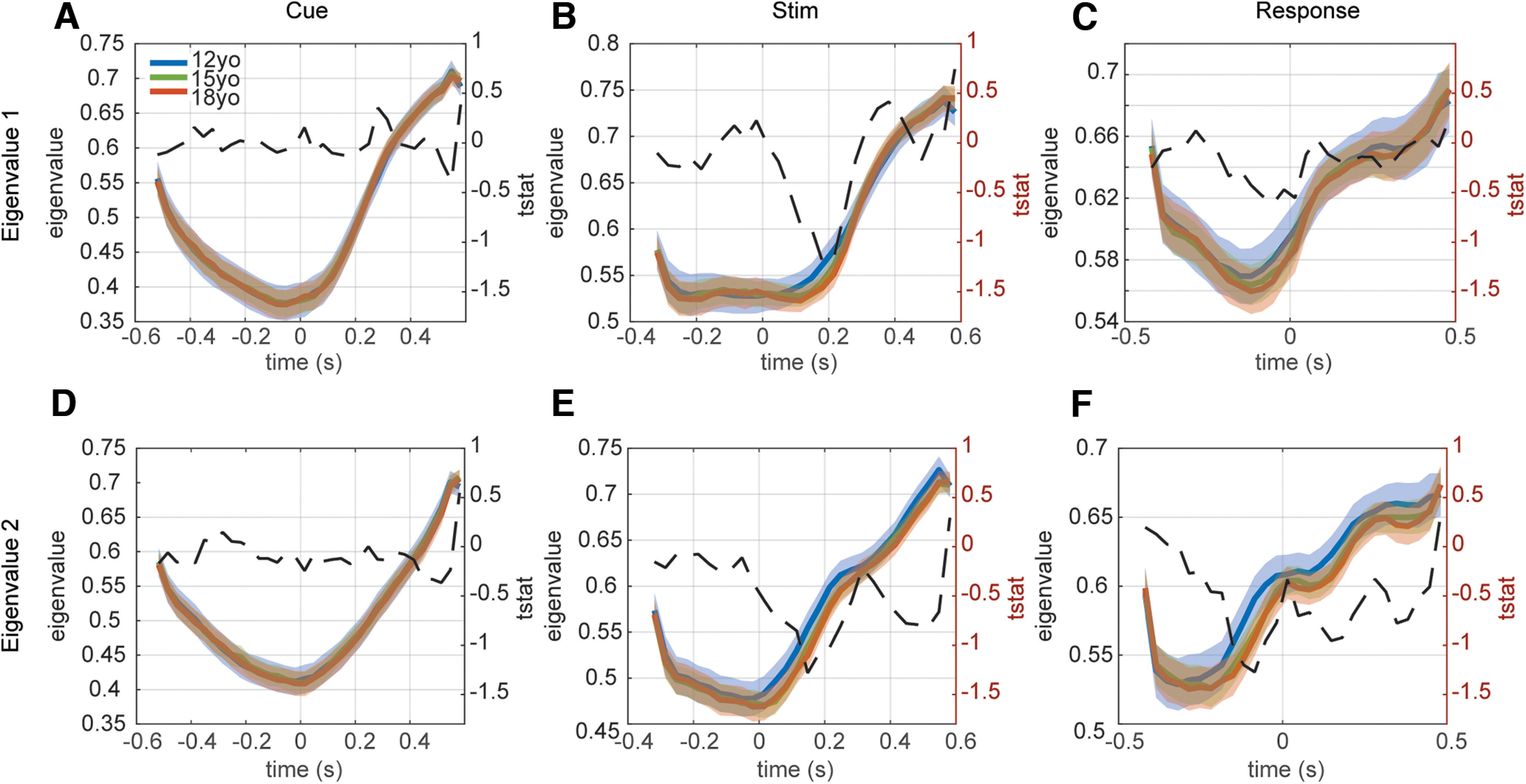
Results of randomizing time offset of evoked response. Panels ***A–F*** show eigenvalues (in solid lines, with shaded SE over subjects) and *t* statistics (in dashed lines) for Cue, Stim, and Response locked analyses of jittered datasets.

We also conducted a control analysis, to examine whether possible differences in the timing of the ERPs across age could account for some of our effects. In analyses locked to the response, we controlled for differences in reaction time. However, to further examine the effect of differences in reaction time, we generated a jittered dataset by assuming that the ERP response on single trials was jittered relative to the mean ERP across trials. The jittered dataset was matched for trials and subjects to the actual dataset. For each subject, we generated a new dataset where the ERP in each single trial was equal to the average ERP for the corresponding condition, but with a time offset given by a sample from the SD of reaction times for the corresponding age group (97, 76, and 72 ms for 12, 15, and 18 years old). We then ran these jittered datasets through the same analysis pipeline used to analyze our actual dataset (Fig. 6). Although there were some differences between the groups, they were less pronounced than the differences seen in the actual dataset (Fig. 4). The differences between ages also did not reach statistical significance. Thus, some of the effects seen in the cue or stimulus locked conditions could be driven by variability across trials in processing speed for different subjects, but they likely do not account for a large fraction of our findings.

## Discussion

We examined changes in EEG dynamics across adolescence, while subjects performed the Eriksen Flanker task. We fit time-varying linear dynamical systems models to the variability in EEG data, and we examined coefficients (eigenvalues) extracted from the models. Smaller coefficients are consistent with faster decay dynamics, which are consistent with dynamical attractors with steeper basins. We found that the coefficients were inversely correlated with age around the time of stimulus presentation; other features of neural dynamics showed no relations with age, suggesting a level of specificity in developmental effects. We also found positive correlations between reaction time, across subjects, and the model coefficients. Thus, subjects with faster reaction times had smaller coefficients. Overall, the results of these analyses are consistent with the predictions of a computational model, that suggest that adolescent synaptic pruning leads to increased behavioral performance, and changes in neural dynamics (B.B. Averbeck, 2022).

Adolescence is an important developmental period. Performance on basic perceptual tasks achieve adult levels by late childhood (Leat et al., 2009), while cross modal-plasticity between the visual and auditory systems (Cohen et al., 1999) and the ability to learn in some domains (Johnson and Newport, 1989) decreases after late childhood. However, adult performance on tasks requiring executive control does not appear until late adolescence (Luna et al., 2010; Larsen and Luna, 2018). Consistent with these changes in behavior, important changes occur in the brain. Imaging experiments have shown that gray matter volume peaks in late childhood and decrease throughout adolescence, while white matter continues to increase during adolescence (Giedd et al., 1999; Gogtay et al., 2004; Bethlehem et al., 2022). Detailed studies of postmortem tissue have found that the number of excitatory synapses in cortex peaks in late childhood and decreases during adolescence (Huttenlocher, 1979; Huttenlocher and Dabholkar, 1997; Petanjek et al., 2011). Some studies suggest that synaptic pruning in prefrontal cortex occurs later than pruning in unimodal sensory areas (Huttenlocher and Dabholkar, 1997; Elston et al., 2009). Furthermore, myelination increases throughout adolescence (Miller et al., 2012). While it is possible that the changes in gray matter volume measured with structural MR are related to the changes in spines in cortex, spines only make up a small fraction (∼1–2%) of the gray matter volume (Rakic et al., 1994), and therefore, this link is not clear. It is possible that synaptic pruning also leads to a decrease in axonal density and dendritic complexity, and these changes contribute to the change in gray matter volume, but this has not, to our knowledge, been investigated. It is also possible that changes in myelination lead to changes in gray matter volume measured with MR, as increased axonal caliber could lead to a change in the MR signal in deep layers of cortex, that is interpreted as a change in gray matter volume (Paus et al., 2008).

Resting state EEG and MEG studies have also found that there are decreases in low-frequency (i.e., θ and α) oscillations and increases in high-frequency (i.e., γ) oscillations (Whitford et al., 2007; Segalowitz et al., 2010). These changes have also been found to correlate with changes in gray matter volume (Whitford et al., 2007; Candelaria-Cook et al., 2022). These findings are broadly, although not completely, consistent with our findings. Most importantly, we examined changes in neural dynamics associated with a behavioral task, and not resting state activity. Moreover, we examined specific neural dynamic parameters linked to pruning through our computational model. Of note, our linear dynamical systems model is a first-order (i.e., AR1) low pass filter. In the extreme case, an eigenvalue of 1 in our model would be an integrator, which is a low-pass filter, and an eigenvalue of 0 would be no filtering on a white noise process. Thus, intermediate values correspond to different amounts of low-pass filtering. Also, relations between age and eigenvalues were selective. We found age-related changes in dynamics during specific task epochs, during which specific cognitive operations were unfolding and behaviors were being generated. We found no such correlations during the initial cue period, that unfolds before behaviors manifest. Because there are limited evoked responses during this period, this analysis would be similar to analyzing resting state activity. However, because of the task structure, we did not have sufficient extratask data to estimate resting-state activity. Regardless, our prior work made no predictions linking pruned computational models to resting-state dynamics (B.B. Averbeck, 2022).

We have focused our interpretation, and the corresponding computational modeling, on pruning. Pruning is a well-known computational approach to make large artificial neural networks learn efficiently with less training data (Le Cun et al., 1990; Bishop, 2006). However, it is difficult to measure pruning in children noninvasively, and we examined no direct measure of pruning in the current study. It will be important in future work to attempt to relate changes in synaptic density over adolescence directly to changes in behavior and neural dynamics using animal models, where pruning can be measured directly (Bourgeois et al., 1994; Chung et al., 2017). EEG scalp potentials are thought to be primarily generated by postsynaptic currents form pyramidal neurons, including currents driven by both excitatory and inhibitory synapses. Pruning is thought to affect only excitatory synapses (Petanjek et al., 2011). Other anatomic changes that occur during adolescence, such as myelination, likely shape behavior and corresponding EEG dynamics. Myelination would likely affect conduction speed and perhaps reliability of transmission (Sugio et al., 2023). The effects of myelination on neural dynamics are less clear, as the effects of conduction delays in nonlinear recurrent networks are complex, and their effects on computation are also unclear. Other physiological changes contributing to EEG signal variability, such as changes in skin conductance with development, are also possible confounding factors. In addition, there are numerous other developmental changes. It will, therefore, be important in future work to continue to consider the effects of these alternative mechanisms, and to carry out additional experiments in animal models, that will allow a more detailed examination of the mechanisms giving rise to these changes. A further limitation of our study is the lack of direct measures of brain anatomy changes in the sample.

### Limitations

We have conducted our analysis in a principal components (PC) space. PCA extracts components that maximize explained variance, but these components are not necessarily biologically relevant (Delorme et al., 2012). Other approaches to dimensionality reduction could have been used, and future work could compare PCA to other approaches to see which might lead to the most consistent results.

In conclusion, we tested hypotheses from a computational model using changes in task-related variability of EEG signals in a longitudinal sample spanning early to late adolescence. We found, using time-dependent linear dynamical systems models, that the EEG signals showed faster decay dynamics in older subjects. Older subjects also had faster reaction times and increased accuracy, and reaction time correlated with eigenvalues. These results extend computational models that use simulated data. Our data and the associated models suggest that adolescent synaptic pruning leads to increased task performance and steeper attractor basins in recurrent networks trained to perform cognitive tasks. The steeper attractor basins predict the changes in eigenvalues over adolescence in the EEG data. Thus, this suggests that synaptic pruning during adolescence may play a fundamental role in the development of adult competence on tasks requiring executive function, including response inhibition, as measured here with the Eriksen Flanker task.

## References

[B1] Averbeck B, O'Doherty JP (2022) Reinforcement-learning in fronto-striatal circuits. Neuropsychopharmacology 47:147–162. 10.1038/s41386-021-01108-0 34354249 PMC8616931

[B2] Averbeck BB (2022) Pruning recurrent neural networks replicates adolescent changes in working memory and reinforcement learning. Proc Natl Acad Sci U S A 119:e2121331119. 10.1073/pnas.2121331119 35622896 PMC9295803

[B3] Averbeck BB, Costa VD (2017) Motivational neural circuits underlying reinforcement learning. Nat Neurosci 20:505–512. 10.1038/nn.4506 28352111

[B4] Benasich A, Ribary U (2018) Emergent brain dynamics: prebirth to adolescence. Cambridge: The MIT Press.

[B5] Bethlehem RAI, et al. (2022) Brain charts for the human lifespan. Nature 604:525–533. 10.1038/s41586-022-04554-y 35388223 PMC9021021

[B6] Bishop CM (2006) Pattern recognition and machine learning. New York: Springer. [Database]

[B7] Blakemore SJ (2018) Inventing ourselves: the secret life of the teenage brain, Ed 1. New York: PublicAffairs.

[B8] Bourgeois JP, Goldman-Rakic PS, Rakic P (1994) Synaptogenesis in the prefrontal cortex of rhesus monkeys. Cereb Cortex 4:78–96. 10.1093/cercor/4.1.78 8180493

[B9] Buzzell GA, Troller-Renfree SV, Barker TV, Bowman LC, Chronis-Tuscano A, Henderson HA, Kagan J, Pine DS, Fox NA (2017) A neurobehavioral mechanism linking behaviorally inhibited temperament and later adolescent social anxiety. J Am Acad Child Adolesc Psychiatry 56:1097–1105. 10.1016/j.jaac.2017.10.007 29173744 PMC5975216

[B10] Caballero A, Granberg R, Tseng KY (2016) Mechanisms contributing to prefrontal cortex maturation during adolescence. Neurosci Biobehav Rev 70:4–12. 10.1016/j.neubiorev.2016.05.013 27235076 PMC5074870

[B11] Candelaria-Cook FT, Solis I, Schendel ME, Wang YP, Wilson TW, Calhoun VD, Stephen JM (2022) Developmental trajectory of MEG resting-state oscillatory activity in children and adolescents: a longitudinal reliability study. Cereb Cortex 32:5404–5419. 10.1093/cercor/bhac023 35225334 PMC9712698

[B12] Chung DW, Wills ZP, Fish KN, Lewis DA (2017) Developmental pruning of excitatory synaptic inputs to parvalbumin interneurons in monkey prefrontal cortex. Proc Natl Acad Sci U S A 114:E629–E637. 10.1073/pnas.1610077114 28074037 PMC5278439

[B13] Cohen LG, Weeks RA, Sadato N, Celnik P, Ishii K, Hallett M (1999) Period of susceptibility for cross-modal plasticity in the blind. Ann Neurol 45:451–460. 10.1002/1531-8249(199904)45:4<451::AID-ANA6>3.0.CO;2-B 10211469

[B14] Collins AGE, Frank MJ (2012) How much of reinforcement learning is working memory, not reinforcement learning? A behavioral, computational, and neurogenetic analysis. Eur J Neurosci 35:1024–1035. 10.1111/j.1460-9568.2011.07980.x 22487033 PMC3390186

[B15] Debnath R, Buzzell GA, Morales S, Bowers ME, Leach SC, Fox NA (2020) The Maryland analysis of developmental EEG (MADE) pipeline. Psychophysiology 57:e13580. 10.1111/psyp.13580 32293719 PMC12758016

[B16] Delorme A, Makeig S (2004) Arnaud Delorme 1, Scott MakeigEEGLAB: an open source toolbox for analysis of single-trial EEG dynamics including independent component analysis. J Neurosci Methods 134:9–21. 15102499 10.1016/j.jneumeth.2003.10.009

[B17] Delorme A, Palmer J, Onton J, Oostenveld R, Makeig S (2012) Independent EEG sources are dipolar. PLoS One 7:e30135. 10.1371/journal.pone.0030135 22355308 PMC3280242

[B18] Elston GN, Oga T, Fujita I (2009) Spinogenesis and pruning scales across functional hierarchies. J Neurosci 29:3271–3275. 10.1523/JNEUROSCI.5216-08.2009 19279264 PMC6666449

[B19] Ferguson HJ, Brunsdon VEA, Bradford EEF (2021) The developmental trajectories of executive function from adolescence to old age. Sci Rep 11:1382. 10.1038/s41598-020-80866-1 33446798 PMC7809200

[B20] Filippi CA, Subar AR, Sachs JF, Kircanski K, Buzzell G, Pagliaccio D, Abend R, Fox NA, Leibenluft E, Pine DS (2020) Developmental pathways to social anxiety and irritability: the role of the ERN. Dev Psychopathol 32:897–907. 10.1017/S0954579419001329 31656217 PMC7265174

[B21] Fox NA, Henderson HA, Rubin KH, Calkins SD, Schmidt LA (2001) Continuity and discontinuity of behavioral inhibition and exuberance: psychophysiological and behavioral influences across the first four years of life. Child Dev 72:1–21. 10.1111/1467-8624.00262 11280472

[B22] Galgali AR, Sahani M, Mante V (2023) Residual dynamics resolves recurrent contributions to neural computation. Nat Neurosci 26:326–338. 10.1038/s41593-022-01230-2 36635498

[B23] Gee DG, Bath KG, Johnson CM, Meyer HC, Murty VP, van den Bos W, Hartley CA (2018) Neurocognitive development of motivated behavior: dynamic changes across childhood and adolescence. J Neurosci 38:9433–9445. 10.1523/JNEUROSCI.1674-18.2018 30381435 PMC6209847

[B24] Giedd JN (2004) Structural magnetic resonance imaging of the adolescent brain. Ann N Y Acad Sci 1021:77–85. 10.1196/annals.1308.009 15251877

[B25] Giedd JN, Blumenthal J, Jeffries NO, Castellanos FX, Liu H, Zijdenbos A, Paus T, Evans AC, Rapoport JL (1999) Brain development during childhood and adolescence: a longitudinal MRI study. Nat Neurosci 2:861–863. 10.1038/13158 10491603

[B26] Gogtay N, Giedd JN, Lusk L, Hayashi KM, Greenstein D, Vaituzis AC, Nugent TF 3rd, Herman DH, Clasen LS, Toga AW, Rapoport JL, Thompson PM (2004) Dynamic mapping of human cortical development during childhood through early adulthood. Proc Natl Acad Sci U S A 101:8174–8179. 10.1073/pnas.0402680101 15148381 PMC419576

[B27] Goldman-Rakic PS (1996) The prefrontal landscape: implications of functional architecture for understanding human mentation and the central executive. Philos Trans R Soc Lond B Biol Sci 351:1445–1453. 8941956 10.1098/rstb.1996.0129

[B28] Huttenlocher PR (1979) Synaptic density in human frontal cortex - developmental changes and effects of aging. Brain Res 163:195–205. 427544 10.1016/0006-8993(79)90349-4

[B29] Huttenlocher PR, Dabholkar AS (1997) Regional differences in synaptogenesis in human cerebral cortex. J Comp Neurol 387:167–178. 10.1002/(SICI)1096-9861(19971020)387:2<167::AID-CNE1>3.0.CO;2-Z 9336221

[B30] Johnson JS, Newport EL (1989) Critical period effects in second language learning: the influence of maturational state on the acquisition of English as a second language. Cogn Psychol 21:60–99. 10.1016/0010-0285(89)90003-0 2920538

[B31] Jonkman LM (2006) The development of preparation, conflict monitoring and inhibition from early childhood to young adulthood: a Go/Nogo ERP study. Brain Res 1097:181–193. 10.1016/j.brainres.2006.04.064 16729977

[B32] Lange-Küttner C, Averbeck BB, Hirsch SV, Wiessner I, Lamba N (2012) Sequence learning under uncertainty in children: self-reflection vs. self-assertion. Front Psychol 3:127. 10.3389/fpsyg.2012.00127 22563324 PMC3342618

[B33] Lange-Küttner C, Averbeck BB, Hentschel M, Baumbach J (2021) Intelligence matters for stochastic feedback processing during sequence learning in adolescents and young adults. Intelligence 86:101542. 10.1016/j.intell.2021.101542

[B34] Larsen B, Luna B (2018) Adolescence as a neurobiological critical period for the development of higher-order cognition. Neurosci Biobehav Rev 94:179–195. 10.1016/j.neubiorev.2018.09.005 30201220 PMC6526538

[B35] Leat SJ, Yadav NK, Irving EL (2009) Development of visual acuity and contrast sensitivity in children. J Optom 2:19–26. 10.3921/joptom.2009.19

[B36] Le Cun Y, Denker JS, Solla SA (1990) Optimal brain damage. In: Advances in neural information processing systems, pp 598–605. Vancouver: Morgan Kaufmann.

[B37] Luna B, Padmanabhan A, O'Hearn K (2010) What has fMRI told us about the development of cognitive control through adolescence? Brain Cogn 72:101–113. 10.1016/j.bandc.2009.08.005 19765880 PMC2815087

[B38] Miller DJ, Duka T, Stimpson CD, Schapiro SJ, Baze WB, McArthur MJ, Fobbs AJ, Sousa AM, Sestan N, Wildman DE, Lipovich L, Kuzawa CW, Hof PR, Sherwood CC (2012) Prolonged myelination in human neocortical evolution. Proc Natl Acad Sci U S A 109:16480–16485. 10.1073/pnas.1117943109 23012402 PMC3478650

[B39] Mognon A, Jovicich J, Bruzzone L, Buiatti M (2011) ADJUST: an automatic EEG artifact detector based on the joint use of spatial and temporal features. Psychophysiology 48:229–240. 10.1111/j.1469-8986.2010.01061.x 20636297

[B40] Nussenbaum K, Hartley CA (2019) Reinforcement learning across development: what insights can we draw from a decade of research? Dev Cogn Neurosci 40:100733. 10.1016/j.dcn.2020.100832 31770715 PMC6974916

[B41] Paus T, Keshavan M, Giedd JN (2008) Why do many psychiatric disorders emerge during adolescence? Nat Rev Neurosci 9:947–957. 10.1038/nrn2513 19002191 PMC2762785

[B42] Petanjek Z, Judaš M, Šimic G, Rasin MR, Uylings HB, Rakic P, Kostovic I (2011) Extraordinary neoteny of synaptic spines in the human prefrontal cortex. Proc Natl Acad Sci U S A 108:13281–13286. 10.1073/pnas.1105108108 21788513 PMC3156171

[B43] Rakic P, Bourgeois JP, Goldman-Rakic PS (1994) Synaptic development of the cerebral cortex: implications for learning, memory, and mental illness. Prog Brain Res 102:227–243. 10.1016/S0079-6123(08)60543-9 7800815

[B44] Rempe MP, Ott LR, Picci G, Penhale SH, Christopher-Hayes NJ, Lew BJ, Petro NM, Embury CM, Schantell M, Johnson HJ, Okelberry HJ, Losh KL, Willett MP, Losh RA, Wang YP, Calhoun VD, Stephen JM, Heinrichs-Graham E, Kurz MJ, Wilson TW (2023) Spontaneous cortical dynamics from the first years to the golden years. Proc Natl Acad Sci U S A 120:e2212776120. 36652485 10.1073/pnas.2212776120 PMC9942851

[B45] Segalowitz SJ, Santesso DL, Jetha MK (2010) Electrophysiological changes during adolescence: a review. Brain Cogn 72:86–100. 10.1016/j.bandc.2009.10.003 19914761

[B46] Smith SM, Nichols TE (2009) Threshold-free cluster enhancement: addressing problems of smoothing, threshold dependence and localisation in cluster inference. Neuroimage 44:83–98. 10.1016/j.neuroimage.2008.03.061 18501637

[B47] Sugio S, Kato D, Wake H (2023) Myelinated axon as a plastic cable regulating brain functions. Neurosci Res 187:45–51. 10.1016/j.neures.2022.11.002 36347403

[B48] Tamnes CK, Walhovd KB, Torstveit M, Sells VT, Fjell AM (2013) Performance monitoring in children and adolescents: a review of developmental changes in the error-related negativity and brain maturation. Dev Cogn Neurosci 6:1–13. 10.1016/j.dcn.2013.05.001 23777674 PMC6987843

[B49] Theodoraki TE, McGeown SP, Rhodes SM, MacPherson SE (2020) Developmental changes in executive functions during adolescence: a study of inhibition, shifting, and working memory. Br J Dev Psychol 38:74–89. 10.1111/bjdp.12307 31587347

[B50] Whitford TJ, Rennie CJ, Grieve SM, Clark CR, Gordon E, Williams LM (2007) Brain maturation in adolescence: concurrent changes in neuroanatomy and neurophysiology. Hum Brain Mapp 28:228–237. 10.1002/hbm.20273 16767769 PMC6871488

[B51] Yoo AH, Collins AGE (2022) How working memory and reinforcement learning are intertwined: a cognitive, neural, and computational perspective. J Cogn Neurosci 34:551–568. 10.1162/jocn_a_01808 34942642

